# Subjective Well-Being by Partnership Status and Its Dependence on the Normative Climate

**DOI:** 10.1007/s10680-012-9257-2

**Published:** 2012-03-30

**Authors:** Ellen Verbakel

**Affiliations:** Department of Sociology, Tilburg University, P.O. Box 90153, 5000 LE Tilburg, The Netherlands

**Keywords:** Well-being, Partnership status, Marriage, Divorce, Multilevel analysis, European Values Study, Bien-être, Statut d’union, Mariage, Divorce, Analyse multi-niveaux, European Values Study

## Abstract

This study first examines the relationship between partnership status and subjective well-being in 45 European countries by analyzing the European Values Study 2008. It was expected and empirically confirmed that married individuals have the highest level of well-being, followed by (in order) cohabiting, dating, single, and finally widowed and divorced individuals. In addition, this study examines to what extent the well-being gaps depend on the normative climate in which an individual lives. It is hypothesized that: (a) being in a non-married relationship (especially cohabitation and divorce) lowers well-being compared to being married in societies that reject non-traditional partnership statuses; and (b) not having a partner is especially detrimental for well-being levels in familialistic societies, which emphasize the importance of a strong, close-knit family. The normative climate appears to hardly affect well-being gaps between partnership statuses. Only the gap between divorced and married women is significantly wider in familialistic societies. It is concluded that the weak dependence of well-being on the normative climate may point at high autonomy in private, relationship-related decisions.

## Résumé

Cette étude examine les relations entre statut d’union et le bien-être subjectif dans 45 pays européens à partir des données de l’Etude Européenne sur les Valeurs de 2008. Il était prévu et confirmé empiriquement que les individus mariés présentent le plus haut niveau de bien-être suivi (en ordre décroissant) par les cohabitants, les personnes dans une relation stable, les célibataires et finalement les veufs et les divorcés. De plus, cet article étudie dans quelle mesure les différences de bien-être dépendent du contexte normatif dans lequel vit l’individu. Les hypothèses sont les suivantes: (a) être dans une situation extra-maritale (plus particulièrement être cohabitant ou divorcé) diminue le bien-être par rapport aux individus mariés dans des sociétés qui rejettent les statuts d’union non-traditionnels (b) ne pas avoir de partenaire est particulièrement préjudiciable au niveau de bien-être dans les sociétés qui valorisent la famille et mettent l’accent sur l’importance d’une famille forte et unie. Les résultats montrent que le contexte normatif a peu d’influence sur les différences de niveaux de bien-être par statut d’union. Dans les sociétés qui valorisent la famille, seule la différence entre femmes divorcées et femmes mariées est significative. En conclusion, le fait que le bien-être dépend peu du climat normatif témoignerait d’une grande autonomie dans les décisions privées relatives à la relation au sein du couple.

## Introduction

In contemporary modern society, the choice regarding how to live one’s life is generally believed to be a free choice. Marriage has long been the standard, but alternative arrangements such as cohabitation, divorce, and same-sex relationships have gained popularity and have increasingly become accepted (Bachrach et al. 2000; Cherlin 2004). Nevertheless, family norms are not equally approving of each partnership type, and these family norms differ between societal contexts. This study investigates (a) to what extent the subjective well-being of individuals differs depending on their partnership status; (b) whether these well-being gaps differ between 45 European countries; and (c) whether the normative climate influences the level of subjective well-being derived from a particular partnership status. Subjective well-being will be defined as life satisfaction.

Early studies on marital status and subjective well-being have mainly focused on the difference between married and non-married individuals (Coombs 1991). Later studies acknowledged that the group of non-married persons is too diverse and should be refined (Soons et al. 2009; Stack and Eshleman 1998). This study simultaneously distinguishes singleness, dating (defined as being in a steady relationship without living together), cohabitation, marriage, divorce, and widowhood, doing justice to the large variability in household arrangements nowadays. More importantly, it offers the opportunity to test whether the resources explanation that is usually offered for the positive relationship between marriage and well-being is also useful for understanding differences in subjective well-being depending on other partnership statuses. Finally, it enables a test of my claim that ‘legal marital status’, which usually only distinguishes between marriage, widowhood, divorce, and never having been married, is a less ideal measure in studies on subjective well-being than ‘current partnership status’, which covers the more extensive list mentioned above. For example, persons registered as widowed or divorced may have found a new partner, resulting in more well-being compared to those whose current partnership status is still widowed or divorced (Mastekaasa 1994).

This study’s country-comparative approach provides a description of country differences in well-being gaps between partnership statuses, and it reveals to what extent societies’ normative contexts are responsible for these gaps. In line with previous large country-comparative studies on the moderating effect of normative climates, I consider disapproval of non-traditional partnership statuses and the importance attached to family support as defining societies’ normative climates. Since these earlier studies are restricted to comparing the well-being of cohabiting and married (Diener et al. 2000; Soons and Kalmijn 2009), or divorced and married individuals (Diener et al. 2000; Kalmijn 2010), studying the complete set of partnership statuses provides a more extensive test of the normative context hypothesis. Although the theoretical ideas in these studies are largely similar, the measures used differ, as do the results. Recent data from the European Values Study 2008 (EVS 2010) have not been used before to study this issue. This dataset provides highly comparable information regarding the values across the European continent, ensuring a substantial variety in normative climates and allowing for the empirical testing of the degree to which the macro context moderates individual-level relationships.

## Theory

### Expected Differences in Well-Being Depending on Partnership Status

The literature offers several causal explanations for why married people can be expected to have higher levels of subjective well-being, as well as a better physical and mental health. Firstly, marriage increases economic resources due to economies of scale and the possibility to pool incomes. Economic resources in turn enhance well-being (Diener et al. 1999; Ross et al. 1990; Stack and Eshleman 1998). Secondly, marriage implies social support in terms of direct help by the partner or access to the partner’s network; living alone would increase the odds of social isolation, which harms one’s sense of belonging and security (Ross et al. 1990; Stack and Eshleman 1998). Thirdly, a spouse offers emotional support. Humans need affection, and having a partner makes people feel cared for, esteemed, loved, and valued as a person (Diener et al. 1999; Ross et al. 1990; Stack and Eshleman 1998). The emotional gratification that results from continuous companionship forms a buffer against the stress of daily life (Coombs 1991; Gove et al. 1990; Kessler and Essex 1982).

Although the above explanations are generally meant to explain why marriage enhances well-being, they essentially differentiate between having a partner or not. Consequently, I argue that these arguments do not exclusively apply to marriage, but to all partnership types involving a partner. On the basis of these general assumptions regarding well-being, it can therefore be assumed that married, cohabiting and dating persons have higher levels of well-being than single, divorced, or widowed persons. However, the explanations concerning economic, social, and emotional resources also allow for more specific speculations about the level of well-being according to different partnership statuses. In the following section, I will derive further expectations about differences in well-being by making distinctions within the two groups of partnership statuses—those that involve a partner and those that do not—in order to predict a rank-ordering of the level of well-being.

The first distinction is made within the group involving a partner: marriage, cohabitation, and dating. Since the argument about sharing and pooling resources predominantly applies to partners who share a household, daters can be expected to have the lowest level of subjective well-being. In addition, partnerships differ in the level of commitment (Kamp Dush and Amato 2005). Commitment may refer to the intensity of the emotional bond and to a long-term time horizon with accompanying relation-specific investments and securities. As a result, commitment ensures both emotional gratifications and economic and social resources. Moreover, uncertainty decreases with rising levels of commitment, and the resulting sense of security contributes to well-being as well (Soons et al. 2009). Marriage is the strongest form of commitment as married partners have proclaimed their intention to share their life forever. Cohabiters have at least expressed their willingness to share a household, demonstrating a higher level of commitment than those at a dating stage. As a result, the lowest level of well-being is expected among dating persons, followed by cohabiting persons, and married persons.

The second distinction is made within the group of partnership statuses that do not involve a partner: being single, divorced, or widowed. In contrast to singles, who have never been in a serious relationship, divorced and widowed people have experienced a negative life event that is usually accompanied by stress and sadness (Mastekaasa 1994). Although it is possible that the negative effect of losing a partner decreases after a while, or that a divorce was experienced as a relief, negative life events are likely to reduce subjective well-being. Death of a spouse, as well as divorce, implies a process of adjustment to the loss of a partner, of rebuilding one’s life, and of coping with the loss of resources (Kitson et al. 1989). The resources explanation leads to the expectation that the negative impact on well-being is stronger for divorced than for widowed persons. Firstly, the economic consequences in case of a break-up are generally worse than those in case of the death of a spouse. Divorce involves dividing up a house and other possessions, which is not the case for widowhood. On top of that, people are often financially protected for the event of widowhood by means of life insurances and widow’s funds; divorce generally implies paying alimony for some and being dependent on alimony for others, and alimentation arrangements after divorce appear not always to be properly executed (McLanahan and Sandefur 1994). Secondly, social resources can be expected to be lower for divorced than for widowed persons because the normative judgement from the outside world can be more disapproving of divorce than of death of a spouse (McLanahan and Sandefur 1994). As a consequence, network members are inclined to offer more support to widowed than to divorced persons. I expect that in the group without a partner, the highest level of well-being will be observed among single individuals, followed by widowed individuals, and lowest level among divorced individuals. In sum, the predicted rank-order from highest to lowest levels of well-being is as follows: married, cohabiting, dating, single, widowed, and divorced.

### The Impact of the Normative Climate

I will examine the impact of two cultural conditions that may moderate the well-being gap between certain partnership statuses. First, an individual’s well-being is expected to be lower if s/he faces disapproval in the environment (Diener et al. 2000; Kalmijn 2010; Soons and Kalmijn 2009). Deviation from social expectations results in a social stigma and produces stress, which in turn reduces subjective well-being. The traditional standard for relationships is marriage; hence the general hypothesis is that the level of well-being will be lower for individuals with all partnership statuses that deviate from this standard. A more refined hypothesis is that the rejection-mechanism will apply most strongly to those individuals with partnership statuses that express explicit and ‘deliberate’ deviation from the standard marriage pattern: cohabitation and divorce. In addition, the argument might apply to singlehood as well, especially when it concerns persons above the age of 35 as these individuals are generally expected to be in a serious relationship by then (cf. the idea of an age deadline for parenthood in Mynarska 2010). Widowhood is not likely to be condemned as it is considered to happen to people and does not result from choice. Dating is usually seen as a normal, non-deviating stage in one’s relationship career and will therefore not be strongly rejected, even in societies that disapprove of non-traditional partnership statuses. As a result, it can be expected that especially the gap in well-being between married persons on the one hand and cohabiting, divorced, and (older) single persons on the other hand will be larger in societies that reject non-traditional partnership statuses. Evidence in support of this hypothesis was found with respect to the well-being gap between cohabiting and married persons (Diener et al. 2000; Soons and Kalmijn 2009), but not for the gap between married and divorced persons in studies that use the normative rejection of divorce as a measurement of the normative climate (Diener et al. 2000; Kalmijn 2010).

The impact of societies’ rejection of non-traditional partnerships can also be expected to become apparent in the comparison of other groups. Cohabiting and dating persons both have partners, but the outside world might condemn cohabiting persons more because they have explicitly made an objectionable choice, whereas daters still have the option to marry directly as one is supposed to do in a traditional view. A similar argument can be made when comparing divorced and single people. Both types of people share the actual state of not having a partner, but the former type have made an explicit objectionable decision from a traditional point of view, whereas the latter have not. Extending the rejection hypothesis leads to the expectation that cohabiters have lower levels of well-being compared to daters and that divorcees have lower levels of well-being compared to singles as societies more strongly reject non-traditional partnership statuses.

A second cultural condition I expect to influence well-being gaps between partnership statuses is the degree of familialism. Familialism expresses the norm of a strong family: it is important that family ties are close and that family members are always available for help. Such a normative climate could have an impact in two different ways. On the one hand, it may make people who fail to meet this family norm unhappier compared to those who are married. This will especially hold for those without a partner (single, divorced, and widowed persons), but also for dating persons and to some extent for cohabiting persons, who do not live up this strong family norm. On the other hand, it may relieve the disadvantages of not having a partner as one can trust to receive support from one’s family members (Diener et al. 2000; Kalmijn 2010). In Italy, for example, it is very common that divorced men and women return to their parental home and are offered practical and financial help by their family members (Ongaro et al. 2009). This would reduce the gap between married persons and single, divorced, or widowed persons. Previous research did not support the idea of family support as a buffer against the negative well-being effects of divorce, but this research relies on relatively indirect measures such as the proportion of unmarried adults living with their parents or the distinction between collectivist and individualist countries (Diener et al. 2000; Kalmijn 2010).

Possibly, more can be said about the direction of the impact of familialism by contrasting other partnership statuses. Consider divorced and single persons or divorced and widowed persons. All these individuals share the lack of a partner who can supply resources; hence they all can benefit from family support. If familialism predominantly stands for the availability of family support, it should not affect the well-being gaps between these groups of individuals. If familialism mainly expresses the norm of a strong family, it can be expected that the difference in well-being between divorced persons on the one hand and single and widowed persons on the other hand increases as societies become more familialistic because it is the divorced group of individuals who have acted explicitly against the norm of building or keeping a strong family.

## Data

This study analyzes data from the most recent wave of the European Values Study 2008 (EVS 2010). The EVS is a large-scale data collection of values and opinions regarding life, family, work, religion, politics, and society in Europe. The 2008 wave includes 47 European countries (or regions like Northern Ireland and Northern Cyprus). Data have been collected under supervision of a local programme director in each country, but they have been centrally coordinated using strict methodological guidelines in order to guarantee high quality and highly comparable data. For more information on the data collection, I refer to the EVS website (www.europeanvaluesstudy.eu). After an age selection (18–79), the exclusion of respondents with a missing value on either subjective well-being or on partnership status, the exclusion of Azerbaijan (recommended by EVS), and the exclusion of Kosovo (because of its outlier position),^1^ the analytical sample consists of 60,518 respondents in 45 countries.

### Individual-Level Variables

The dependent variable, subjective well-being, is a broad concept often defined as either life satisfaction or happiness. Although the two are highly correlated, they point at different things (Gundelach and Kreiner 2004). Happiness is defined as an emotional response, an experience of affect, whereas life satisfaction refers to a cognitive evaluation of one’s life (Campbell et al. 1976; Diener et al. 1999; Lane 2000). In this study, I define subjective well-being as life satisfaction measured by the question “All things considered, how satisfied are you with your life as a whole these days?” Answers have been given on a ten-point scale with higher scores indicating higher levels of well-being. The average level of well-being is 7 for men and women. Countries vary considerably in average well-being: from 5.5 in Georgia to 8.4 in Denmark.

For descriptive purposes, legal marital status will be distinguished from current partnership status. *Legal marital status* has been asked to respondents directly; answer categories were: married, registered partnership, widowed, divorced, separated, and never married. Registered partnerships (reported by 2 % of the sample) do not exist in all countries and are therefore combined with marriage. Moreover, the categories ‘divorced’ and ‘separated’ (reported by 1.5 % of the sample) have been collapsed. Current *partnership status* has been derived from a more extensive module on current and prior relationships. People who are married or have a registered partnership have been divided into those who are in their first marriage and those who have been married to (or had a registered partnership with) another partner before. Cohabitation refers to living together unmarried with a partner. This partnership status may refer to people who are officially registered as being divorced, widowed, or never married. Dating respondents have indicated to be unmarried and not in a registered partnership, not to live with a partner, but to have a steady relationship. The definition of a ‘steady’ relationship is not provided in the questionnaire and thus reflects the respondent’s evaluation. Again, this partnership status does not refer to an official marital status and can therefore include respondents who have been divorced, widowed, or never married. Respondents have been categorized as single if they have never been married, do not live with a partner and do not have a steady relationship. In an additional test, I distinguish singles below the age of 35 and singles of 35 years and older to find out whether older singles are more sensitive to societal disapproval of not being married. Current partnership status is classified as divorced or widowed if the respondent has indicated this relationship type as his/her legal marital status (separation after a cohabiting relationship has been considered a divorce), does not live with a partner, and does not have a steady relationship.

The relationship between partnership status and subjective well-being will be controlled for several individual characteristics that have been argued to affect well-being and the likelihood of being in a certain partnership status. They are inspired by common findings in the literature (Diener et al. 1999; Wilson 1967). *Education* has been measured with the international standard coding scheme (ISCED 1-digit) that distinguishes seven levels running from pre-primary or no education (0) to second stage tertiary education (6). The *age* range has been limited to 18 through 79, and age squared has been included because the expected decline in well-being when getting older is expected to level off. *Religiousness* is a binary variable that labels someone as a religious person if one belongs to a denomination and attends religious services at least once a month. The *unemployed* have been distinguished from (non-)employment categories because unemployment is often found to negatively affect well-being due to its involuntary character.

Two individual-level explanations for differences in well-being between partnership statuses will be included in the models. *Household income* reflects economic resources available to the respondent. Household incomes have been adjusted for differences in power purchase parities and expressed in Euros (×1,000). *Health* refers to subjective health, retrieved from the question “All in all, how would you describe your state of health these days? Would you say it is very good, good, fair, poor, or very poor?” The answer categories have been reversed so that a higher score means a better health (range from 0 to 4).

Missing values on categorical independent variables have been coded as a separate dummy variable; missing values on continuous independent variables have been imputed by conditional country means.^2^ With the exception of household income (18 %), values were missing in less than 1 % of the cases. The models control for dummy variables indicating the information was originally missing (results not shown). Table 1 presents the descriptive information on the individual characteristics.

**Table 1:** Descriptive information on individual characteristics

	Minimum	Maximum	Males		Females	
			Mean	SD	Mean	SD
Subjective well-being	1	10	7.05	2.27	6.99	2.31
Partnership status						
Married			0.60		0.56	
Cohabiting			0.06		0.06	
Dating			0.07		0.06	
Single			0.19		0.13	
Divorced			0.04		0.07	
Widowed			0.03		0.11	
Legal marital status						
Married			0.60		0.56	
Never married			0.29		0.22	
Widowed			0.04		0.12	
Divorced			0.07		0.10	
Education^a^	0	6	3.14	1.30	3.06	1.39
Age	18	79	45.30	16.71	45.73	16.56
Religious			0.23		0.31	
Unemployed			0.10		0.09	
Household income (ppp ×1,000)^a^	0	14.73	1.38	1.37	1.18	1.24
Health	0	4	2.80	0.92	2.65	0.96
Rejection of non-traditional partnership statuses	0	1	0.41	0.29	0.42	0.29
Familialistic norm	0	1	0.65	0.39	0.63	0.39

*Source*: European Values Study, 2008 (*N* = 60,518 individuals in 45 countries)

^a^Of non-missing observations only

### Country-Level Variables

The first measure of the normative climate is *rejection of non*-*traditional partnership statuses* which is an aggregated measure from individual responses to three items. Respondents scored 1 on the subsequent items if they gave answers 1 through 4 to the question whether they think divorce can always be justified, never be justified, or something in between (scale runs from 1 “never” to 10 “always”); disagree (strongly) with the statement “It is alright for two people to live together without getting married”; and disagree with the statement “Marriage is an outdated institution”. The scores have been averaged so that the scale runs from 0 to 1 with higher scores meaning a stronger rejection of non-traditional partnerships. The countries that are most accepting of non-traditional partnership statuses are Sweden, France, and Luxembourg (0.27), and the population is most traditional with respect to partnership statuses in Turkey (0.68).

*Familialism* represents the norm of a strong family in which family members should be prepared to help each other at whatever costs. This measure has been aggregated from individual responses as well, and is based on two items: (a) “Which of the following statements best describes your views about parents’ responsibilities to their children? 1—Parents’ duty is to do their best for their children even at the expense of their own well-being, 2—Parents have a life of their own and should not be asked to sacrifice their own well-being for the sake of their children”; (b) “Which of the following statements best describes your views about responsibilities of adult children towards their parents when their parents are in need of long-term care? 1—Adult children have the duty to provide long-term care for their parents even at the expense of their own well-being, 2—Adult children have a life of their own and should not be asked to sacrifice their own well-being for the sake of their parents”. Those who answer that family members have a duty to help each other in both cases receive score 1; those who give this answer only in one of the two cases receive score 0.5; those who find that both parents and children should not be asked to sacrifice their own well-being receive score 0. Familialism is lowest in Finland (0.33) and highest in Georgia (0.90).

As will be explained in more detail in the next section, I will account for possible selectivity of the married group in each country (Kalmijn 2010; Stack and Eshleman 1998). Average well-being of the group of married people depends on how easy it is to leave an unhappy marriage or to choose another living arrangement. For each country, I calculated the proportion of persons that ever divorced and ever cohabited. These proportions have been standardized over countries and then averaged. High values imply that the group of married people is relatively selective, and can therefore be expected to have a relatively high level of well-being. The highest value is found in Denmark (2.22), the lowest value in Turkey (−1.43). Descriptive information on the country-level variables can be found in Table 2. For the distribution of partnership statuses by country, see Appendix.

**Table 2:** Descriptive information on country characteristics

	Average subjective well-being	Rejection of non-traditional partnership statuses	Familialism	Selectivity of married group	*N*
Albania	6.36	0.49	0.72	−1.22	1,500
Armenia	5.69	0.64	0.83	−1.27	1,389
Austria	7.55	0.31	0.45	0.54	1,453
Belarus	6.11	0.41	0.56	0.00	1,456
Belgium	7.62	0.31	0.60	0.47	1,460
Bosnia Herzegovina	7.05	0.52	0.81	−1.16	1,401
Bulgaria	5.76	0.40	0.68	−0.48	1,380
Croatia	7.12	0.48	0.73	−0.53	1,338
Cyprus	7.30	0.51	0.70	−0.41	949
Czech Republic	7.19	0.38	0.49	0.62	1,674
Denmark	8.38	0.32	0.42	2.22	1,438
Estonia	6.66	0.38	0.57	1.11	1,434
Finland	7.67	0.31	0.33	1.86	1,095
France	7.10	0.27	0.67	1.29	1,399
Georgia	5.47	0.63	0.90	−1.29	1,456
Germany	6.78	0.33	0.49	1.20	1,975
Great Britain	7.52	0.34	0.56	0.86	1,403
Greece	6.87	0.38	0.74	−0.59	1,407
Hungary	6.34	0.37	0.65	0.33	1,461
Iceland	8.04	0.31	0.52	1.38	763
Ireland	7.79	0.38	0.53	−0.48	889
Italy	7.18	0.44	0.71	−0.88	1,339
Latvia	6.39	0.41	0.55	0.82	1,392
Lithuania	6.41	0.37	0.37	0.14	1,426
Luxembourg	7.86	0.27	0.60	0.43	1,578
Macedonia	6.87	0.47	0.69	−1.16	1,362
Malta	7.87	0.63	0.83	−1.40	1,422
Moldova	6.55	0.57	0.78	−0.41	1,483
Montenegro	7.46	0.45	0.73	−0.98	1,464
Netherlands	7.98	0.32	0.53	0.42	1,413
Northern Cyprus	6.30	0.53	0.69	−0.86	480
Northern Ireland	7.89	0.39	0.52	−0.26	437
Norway	8.10	0.32	0.55	1.66	1,086
Poland	7.26	0.47	0.68	−0.45	1,394
Portugal	6.51	0.34	0.83	−0.60	1,408
Romania	6.79	0.43	0.69	−0.38	1,393
Russian Federation	6.51	0.41	0.58	0.55	1,395
Serbia	6.96	0.44	0.66	−0.55	1,449
Slovak Republic	7.12	0.51	0.60	−0.59	1,347
Slovenia	7.57	0.36	0.67	−0.66	1,256
Spain	7.33	0.29	0.74	−0.26	1,379
Sweden	7.72	0.27	0.51	1.20	1,037
Switzerland	8.01	0.30	0.56	1.13	1,161
Turkey	6.50	0.68	0.81	−1.43	2,256
Ukraine	6.04	0.49	0.70	0.08	1,441
Mean	7.06	0.41	0.63	0.00	
SD	0.71	0.11	0.13	0.96	
Minimum	5.47	0.27	0.33	−1.43	
Maximum	8.38	0.68	0.90	2.22	

*Source*: European Values Study, 2008 (*N* = 45)

## Descriptive Results

In this section, I will answer the descriptive questions to what extent subjective well-being varies by partnership status and to what extent well-being gaps between partnership statuses vary over countries.

### Subjective Well-Being by Partnership Status

In order to better understand the relationship between partnership status and subjective well-being, special attention will be paid to the issue of using legal marital status rather than current partnership status as a measure. The latter takes into account that widowed or divorced persons may have met a new partner (without being married) or that never married persons may actually be in a serious relationship. Analyses will be performed for men and women separately because previous research suggests that partnership affects well-being differently for men than women (Coombs 1991). Tables 3 and 4 show the average well-being levels by legal marital status and current partnership status for men and women, respectively. The scores have been controlled for education, age, age squared, religiosity, and unemployment and are derived from random intercept models, implying that country differences in the intercept of subjective well-being are taken into account. Note that the number of cases in these tables is slightly lower than in the multilevel analyses that follow because for some respondents not enough information was available to combine their legal marital status and current partnership status.

**Table 3:** Subjective well-being of males by legal marital status and current partnership status, controlled for education, age, age^2^, religiosity, and unemployment in random intercept model

Males	Current partnership status							
Legal marital status	Married, 1st time	Remarried	Cohabiting	Dating	Single	Widowed	Divorced	Average well-being
Married/registered partnership	7.37	7.35						7.37
Never married			7.08	7.02	6.70			6.82
Widowed			7.37	7.17		6.29		6.42
Divorced/separated			7.24	7.00			6.44	6.69
Average well-being	7.37	7.34	7.13	7.02	6.71	6.29	6.44	7.12

*Source*: European Values Study, 2008 (*N* = 26,414 in 46 countries)

Well-being scores apply to non-religious, employed men with average education and age

Differences within rows and within columns significant at 5 % level, except for: (1) Comparisons within rows: married 1st time vs. remarried; never married, cohabiting vs. never married, dating; widowed, cohabiting vs. widowed, dating; divorced, cohabiting vs. divorced, dating; average well-being: married 1st time vs. remarried; cohabiting vs. dating; widowed vs. divorced. (2) Comparisons within columns: never married, cohabiting vs. widowed, cohabiting vs. divorced, cohabiting; never married, dating vs. widowed, dating vs. divorced, dating

**Table 4:** Subjective well-being of females by legal marital status and current partnership status, controlled for education, age, age^2^, religiosity, and unemployment in random intercept model

Females	Current partnership status							
Legal marital status	Married, 1st time	Remarried	Cohabiting	Dating	Single	Widowed	Divorced	Average well-being
Married/registered partnership	7.35	7.24						7.34
Never married			7.04	6.81	6.77			6.83
Widowed			7.17	7.23		6.54		6.60
Divorced/separated			7.03	6.96			6.44	6.59
Average well-being	7.35	7.24	7.05	6.87	6.78	6.54	6.44	7.06

*Source*: European Values Study, 2008 (*N* = 32,835 in 46 countries)

Well-being scores apply to non-religious, employed women with average education and age

Differences within rows and within columns significant at 5 % level, except for: (1) Comparisons within rows: married 1st time vs. remarried; never married, dating vs. never married, single; widowed, cohabiting vs. widowed, dating; divorced, cohabiting vs. divorced, dating; average well-being: married 1st time vs. remarried; dating vs. single; widowed vs. divorced. (2) Comparisons within columns: never married, cohabiting vs. widowed, cohabiting vs. divorced, cohabiting; never married, dating vs. widowed, dating vs. divorced, dating; average well-being: widowed vs. divorced

The first conclusion that can be drawn is that the expected ordering of well-being levels according to partnership status is largely confirmed. Referring to the average levels of well-being by current partnership status (bottom row), married persons form the happiest group. Although men and women in their first marriage seem slightly happier than remarried men and women, this difference is not significant. The group of married persons is followed by cohabiting, dating, and single persons, respectively. Widowed and divorced people report lowest levels of subjective well-being. I expected that divorced people would have lower levels of well-being than widowed people. This is true for women, but not for men. For men, widowhood lowers well-being more strongly than divorce, whereas for women, divorce is more detrimental than widowhood; neither difference reaches the level of significance though. Another interesting difference between men and women is that for men the presence of a partner markedly enhances their well-being, whereas for women it is the sharing a household with a partner that makes the difference. More precisely, subjective well-being does not differ significantly between cohabiting and dating men, but is higher than the well-being of single men. For women, well-being levels do not differ significantly between single and dating women, but are higher for those who cohabit. Overall, the variation in well-being is quite substantial; the difference between the most and least content groups is about one point on a ten-point scale.

The second conclusion is that it makes sense to consider current partnership status instead of legal marital status in studies on subjective well-being. Well-being levels within the groups of persons who are officially registered as widowed, divorced, or never married are higher for those who have found a (new) partner to whom they are not married (as marriage would change their legal marital status). This confirms earlier findings by Mastekaasa (1994). Note that the negative event of loosing or separating from a partner may have occurred longer ago for those who are currently in a new relationship compared to those who have not found a new partner and that time since widowhood or divorce may be the explanation for their higher well-being. This alternative explanation cannot be tested with the data at hand. For both officially widowed and divorced men and women, it does not make a significant difference whether they are dating a new partner or whether they live together. Similar to the earlier observed pattern in the bottom row, never married men report higher levels of well-being if they are dating or cohabiting compared to being single, whereas never married women report only higher levels of well-being only if they are cohabiting; dating and being single produce the same levels of well-being.

In the light of the remainder of this study, it can be concluded that some substantial differences are ignored when legal marital status is used to study the relationship between partnership status and subjective well-being; the groups are rather heterogeneous as their well-being is affected by their current partnership status. When studying the relationship between current partnership status and well-being, Tables 3 and 4 demonstrate that it is not necessary to differentiate by relationship history; within the groups of currently cohabiting or currently dating persons, no significant differences in well-being are observed between the groups of legally divorced, widowed, or never married persons. Because the difference between first marriage and remarriage does not appear to be significant, those groups will be combined in further analyses. The other partnership statuses will be considered separately.

### Variation in Well-Being Gaps Over Countries

**Fig. 1:**
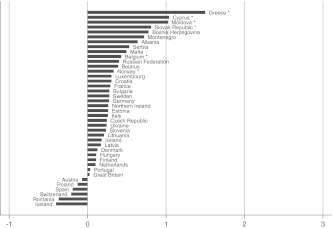
Well-being gap between married and cohabiting people by country. Countries with fewer than 20 persons in the married or cohabiting category have been excluded. *p < 0.05

**Fig. 2:**
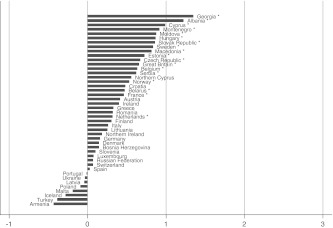
Well-being gap between married and dating people by country. Countries with fewer than 20 persons in the married or dating category have been excluded. *p < 0.05

**Fig. 3:**
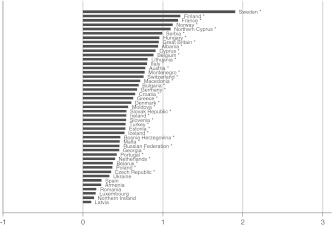
Well-being gap between married and single people by country. Countries with fewer than 20 persons in the married or single category have been excluded**.** *p < 0.05

**Fig. 4:**
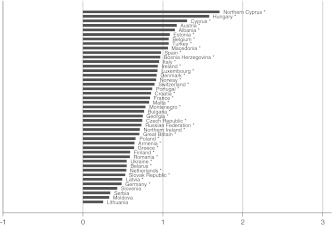
Well-being gap between married and widowed people by country. Countries with fewer than 20 persons in the married or widowed category have been excluded. *p < 0.05

**Fig. 5:**
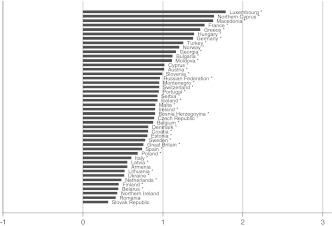
Well-being gap between married and divorced people by country. Countries with fewer than 20 persons in the married or divorced category have been excluded. *p < 0.05

The next step is to examine whether well-being gaps between partnership statuses vary over countries. A descriptive answer can be obtained from Figs. 1, 2, 3, 4, and 5, which depict the differences in well-being between married and all other groups by country. This difference score represents the unstandardized regression coefficient from a single country analysis that is controlled for the individual-level confounding variables. Since in several countries the number of men and women in particular partnership statuses are extremely low, I combined men and women. If the number of cases in a partnership status in a country was below 20, the country is not included in the figure.

The size of the well-being gaps appears to differ substantially between countries, implying that there is some variation to be explained. In some countries married persons report on average about two points (on a 10-point scale) more subjective well-being than others; in other countries the gaps are very small, or married persons tend even to have less well-being than others. Not all gaps reach the level of significance in each country, especially in the case of the married-cohabitating gap. The figures do show, however, that the direction of the gap is generally in favor of the married group, and that the size of the gaps increases on average from Figs. 1, 2, 3, 4, and 5, which is in accordance with the expected order of well-being gaps.

A clear pattern in the country differences is hard to detect at first sight. Besides differences in normative climate as hypothesized earlier, there is another possible explanation for country differences that is related to selectivity of the married group in a country. It can be argued that the more selective the group of married people is, the more well-being they will have; and as a result, the gap with other groups can be relatively large. This selectivity depends on the availability of alternative options: divorce and cohabitation. If a country poses high barriers to divorce (be it legally, normatively, or economically), many unhappy couples will remain together, which reduces the average level of well-being of married people. Similarly, if cohabitation is a common alternative to marriage, the couples who nevertheless choose to marry are likely to be very certain about and satisfied with their relationship, thereby increasing the average level of well-being of married people (Kalmijn 2010; Stack and Eshleman 1998). Countries differ substantially in divorce and cohabitation rates (Kalmijn 2007), and generally it can be expected that in traditional countries (where one expects larger gaps because of the normative rejection and family norm) the group of married people is less selective (which expectedly leads to smaller gaps). In the multivariate analyses, this selectivity issue will be taken into account by including interaction terms between the degree of selectivity of the married group and the partnership statuses.

## Results from Multilevel Analyses

### Models

The data will be analyzed with random intercept multilevel regression models with individuals nested in countries. Random slopes of the partnership statuses are included to assess to what extent the relationship between partnership status and well-being varies over countries. I will conduct analyses separately for men and women. Empty models reveal that the intra-class correlation is 0.24 for men and 0.25 for women implying that a quarter of the variation in subjective well-being can be attributed to differences between countries and three quarters to differences within countries.

Model 1 summarizes the descriptive results from the previous section showing the difference in subjective well-being of cohabiting, dating, single, widowed, and divorced persons compared to married persons net of the impact of control variables. Model 2 includes two important individual-level explanations for well-being differences, household income and health, and reveals how much of the original differences remain. These differences are conditioned on the normative context in the next models. Model 3 tests the dependence of the well-being gaps on the rejection of non-standard family types by including cross-level interactions with partnership status. As explained in the previous section, this model controls for the interactions between the selectivity of the married group in a country and partnership status. In addition, the model controls for each individual’s opinion on non-standard relationship types and its interactions with partnership status to avoid the cross-level interactions to be contaminated by individual mechanisms.^3^ In Model 4 the normative rejection of non-traditional family arrangements is replaced by the norm of a strong family. Since the two value concepts are positively related (*r* = 0.67), both sets of interaction terms are added simultaneously in Model 5, although this means that the number of degrees of freedom becomes relatively small. All individual- and country-level variables are standardized so that score zero refers to the ‘average individual’ or to the ‘average country’.

### Results

Models 1 in Tables 5 and 6 replicate the conclusions from the descriptive results for men and women, respectively. The expected order of partnership statuses by their average level of well-being runs from married, to cohabiting, to dating, to single persons; for men followed by divorced and widowed persons, for women first by widowed then by divorced persons. The differences in well-being between all groups are significant with the exception of the well-being gap between cohabiting and dating men and between single and dating women (as we have seen in the descriptive table before) and between widowed and divorced men. The difference between widowed and divorced women almost reaches conventional levels of significance (*p* = 0.09) as well as the difference between single and divorced men (*p* = 0.06). The well-being gaps can be interpreted on the ten-point life satisfaction scale; they vary from −0.27 for the male married-cohabiting gap to −1.06 for the male married-widowed gap. In terms of effect sizes, the gaps are reasonable, varying from 0.12 for the male married-cohabiting gap to 0.47 for the male married-widowed gap.^4^ Control variables show a common pattern: well-being is higher among highly educated, religious, and employed men and women, and it decreases with age, but this effect levels off over the life course. The bottom panel of the table presents the random slopes of the partnership statuses. Interestingly, it reveals that the slope of cohabitation, representing the difference in well-being between married and cohabiting persons, does not vary over countries.^5^ Although eyeballing Fig. 1 would lead one to expect differently, the fact that the marriage-cohabitation gap only significantly deviates from zero in 6 out of 45 countries makes it less surprising that the slope of cohabitation does not differ significantly over countries. It implies that this well-being gap is not likely to be found to depend on societies’ normative contexts, which will be examined in Models 3 and 4.

**Table 5:** Multilevel analysis on subjective well-being of men

Males	Model 1		Model 2		Model 3^a,b^		Model 4^a,c^		Model 5^a,d^	
	*b*	SE	*b*	SE	*b*	SE	*b*	SE	*b*	SE
*Individual level*										
Married (ref)										
Cohabiting	−0.27**	0.06	−0.19**	0.05	−0.19**	0.07	−0.24**	0.06	−0.20**	0.07
Dating	−0.34**	0.07	−0.26**	0.06	−0.24**	0.07	−0.27**	0.07	−0.24**	0.07
Single	−0.67**	0.07	−0.53**	0.05	−0.54**	0.05	−0.54**	0.05	−0.54**	0.05
Widowed	−1.06**	0.10	−0.85**	0.09	−0.83**	0.09	−0.85**	0.09	−0.84**	0.10
Divorced	−0.90**	0.10	−0.69**	0.08	−0.64**	0.09	−0.67**	0.08	−0.64**	0.09
Household income (×1,000, std.)			0.15**	0.02	0.14**	0.02	0.14**	0.02	0.14**	0.02
Health (std.)			0.76**	0.01	0.76**	0.01	0.76**	0.01	0.76**	0.01
Education (std.)	0.20**	0.02	0.07**	0.01	0.07**	0.01	0.07**	0.01	0.07**	0.01
Age (std.)	−1.26**	0.09	−0.92**	0.09	−0.90**	0.09	−0.92**	0.09	−0.91**	0.09
Age square (std.)	1.03**	0.09	0.97**	0.08	0.95**	0.08	0.97**	0.08	0.96**	0.08
Religious	0.12**	0.03	0.12**	0.03	0.11**	0.03	0.12**	0.03	0.11**	0.03
Unemployed	−0.78**	0.05	−0.62**	0.04	−0.62**	0.04	−0.62**	0.04	−0.62**	0.04
*Country level*										
Rejection of non-traditional partnership statuses (std.)					−0.15	0.10			−0.13	0.11
^×^Cohabiting					−0.05	0.10			−0.06	0.10
^ ×^Dating					0.10	0.10			0.10	0.10
^×^Single					0.05	0.07			0.07	0.07
^ ×^Widowed					−0.09	0.14			−0.05	0.15
^×^Divorced					0.08	0.13			0.10	0.13
Familialism (std.)							−0.12	0.11	−0.09	0.11
^×^Cohabiting							−0.07	0.09	−0.07	0.09
^ ×^Dating							0.05	0.10	0.03	0.10
^ ×^Single							−0.03	0.08	−0.04	0.08
^×^Widowed							−0.22	0.14	−0.21	0.15
^ ×^Divorced							−0.12	0.13	−0.13	0.13
Intercept	7.36**	0.11	7.21**	0.07	7.21**	0.07	7.21**	0.07	7.21**	0.07
Variance individual level	2.11	0.01	2.00	0.01	2.00	0.01	2.00	0.01	2.00	0.01
Variance country level										
Intercept	0.70^#^	0.08	0.46^#^	0.05	0.43^#^	0.05	0.43^#^	0.05	0.43^#^	0.05
Slope cohabitation	0.00	0.00	0.00	0.00	0.00	0.00	0.00	0.00	0.00	0.00
Slope dating	0.21^#^	0.09	0.20^#^	0.08	0.20^#^	0.08	0.20^#^	0.08	0.20^#^	0.08
Slope single	0.40^#^	0.06	0.24^#^	0.05	0.21^#^	0.05	0.21^#^	0.05	0.22^#^	0.05
Slope widowed	0.40^#^	0.10	0.32^#^	0.10	0.34^#^	0.10	0.34^#^	0.09	0.35^#^	0.10
Slope divorced	0.49^#^	0.09	0.35^#^	0.08	0.33^#^	0.08	0.34^#^	0.08	0.34^#^	0.08
*N* respondents	26,996		26,996		26,996		26,996		26,996	
*N* countries	45		45		45		45		45	

*Source*: European Values Study, 2008

* *p* < 0.05, ** *p* < 0.01,^#^ at least twice the standard error

^a^M3–M5 include the degree of selectivity of the married group in the country and its interactions with partnership status

^b^M3 includes individual rejection of non-traditional partnership statuses and its interactions with partnership status

^c^M4 includes individual familialism and its interactions with partnership status

^d^M5 includes both sets of control variables of M3 and M4

**Table 6:** Multilevel analysis on subjective well-being of women

Females	Model 1		Model 2		Model 3^a,b^		Model 4^a,c^		Model 5^a,d^	
	*b*	SE	*b*	SE	*b*	SE	*b*	SE	*b*	SE
*Individual level*										
Married (ref)										
Cohabiting	−0.29**	0.05	−0.22**	0.05	−0.21**	0.06	−0.22**	0.06	−0.21**	0.06
Dating	−0.48**	0.06	−0.39**	0.06	−0.34**	0.06	−0.39**	0.06	−0.34**	0.06
Single	−0.58**	0.07	−0.50**	0.06	−0.49**	0.06	−0.50**	0.06	−0.49**	0.06
Widowed	−0.77**	0.05	−0.63**	0.05	−0.62**	0.05	−0.61**	0.05	−0.62**	0.05
Divorced	−0.91**	0.06	−0.77**	0.06	−0.77**	0.06	−0.78**	0.05	−0.76**	0.06
Household income (×1,000, std.)			0.10**	0.02	0.10**	0.02	0.10**	0.02	0.10**	0.02
Health (std.)			0.76**	0.01	0.76**	0.01	0.76**	0.01	0.76**	0.01
Education (std.)	0.20**	0.01	0.07**	0.01	0.07**	0.01	0.07**	0.01	0.07**	0.01
Age (std.)	−1.11**	0.08	−0.86**	0.08	−0.82**	0.08	−0.84**	0.08	−0.81**	0.08
Age square (std.)	0.88**	0.08	0.91**	0.08	0.86**	0.08	0.88**	0.08	0.85**	0.08
Religious	0.19**	0.03	0.16**	0.03	0.13**	0.03	0.16**	0.03	0.13**	0.03
Unemployed	−0.63**	0.04	−0.52**	0.04	−0.53**	0.04	−0.53**	0.04	−0.53**	0.04
*Country level*										
Rejection of non-traditional partnership statuses (std.)					−0.16	0.11			−0.14	0.11
^×^Cohabiting					0.02	0.09			0.03	0.09
^×^Dating					0.05	0.10			0.04	0.10
^×^Single					0.02	0.08			0.03	0.08
^×^Widowed					0.01	0.07			0.02	0.07
^×^Divorced					0.00	0.09			0.04	0.09
Familialism (std.)							−0.13	0.11	−0.10	0.12
^×^Cohabiting							−0.01	0.08	−0.02	0.08
^×^Dating							0.06	0.10	0.06	0.10
^×^Single							−0.03	0.09	−0.03	0.09
^×^Widowed							−0.09	0.07	−0.09	0.07
^ ×^Divorced							−0.23**	0.08	−0.24**	0.08
Intercept	7.34**	0.12	7.30**	0.08	7.31**	0.07	7.30**	0.07	7.31**	0.07
Variance individual level	2.13	0.01	2.03	0.01	2.02	0.01	2.03	0.01	2.02	0.01
Variance country level										
Intercept	0.76^#^	0.08	0.51^#^	0.06	0.46^#^	0.05	0.46^#^	0.05	0.46^#^	0.05
Slope cohabiting	0.00	0.00	0.00	0.00	0.00	0.00	0.00	0.00	0.00	0.00
Slope dating	0.18	0.10	0.16	0.09	0.18^#^	0.09	0.17	0.09	0.18^#^	0.09
Slope single	0.40^#^	0.06	0.27^#^	0.05	0.26^#^	0.05	0.26^#^	0.05	0.27^#^	0.05
Slope widowed	0.19^#^	0.06	0.15^#^	0.05	0.14^#^	0.05	0.13^#^	0.06	0.14^#^	0.06
Slope divorced	0.27^#^	0.08	0.25^#^	0.07	0.19^#^	0.08	0.16	0.08	0.16	0.08
*N* respondents	33,522		33,522		33,522		33,522		33,522	
*N* countries	45		45		45		45		45	

*Source*: European Values Study, 2008

* *p* < 0.05, ** *p* < 0.01, ^#^ at least twice the standard error

^a^M3–M5 include the degree of selectivity of the married group in the country and its interactions with partnership status

^b^M3 includes individual rejection of non-traditional partnership statuses and its interactions with partnership status

^c^M4 includes individual familialism and its interactions with partnership status

^d^M5 includes both sets of control variables of M3 and M4

Model 2 includes the mediating variables household income and health. Note that to some extent both are preceding variables as well since healthy and rich people are more attractive (marriage) partners. Household income and health both have the expected positive relationship with subjective well-being and explain 20–30 % of the male well-being gaps and 15–24 % of the female well-being gaps. What is interesting is that the differences in well-being between marriage and the other partnership statuses remain substantial and significant. These gaps may be due to differences in countries’ normative climates.

Models 3 and 4 provide the test for the moderating role of the normative context. Is it true that people in a non-married relationship suffer more in terms of well-being in societies that generally disapprove of non-traditional partner statuses? The answer is negative: none of the interaction terms in Model 3 are significant (and they are very small compared to their standard error), indicating that societal rejection of one’s partnership status does not make people unhappier. This holds true for both men and women. The contrasts between dating and cohabiting and between single and divorced persons are not significant either implying another rejection of the hypothesis. Additional analyses (results not shown) revealed that the impact of societal disapproval of non-traditional partnership statuses does not affect older singles (35 years or older) more than younger singles (under age 35). I also tested whether disapproval of divorce (without being part of the larger concept of rejection of non-traditional partnership statuses) would moderate well-being gaps, and I did the same for disapproval of cohabitation. In either case, no significant interaction effects were observed.

For men, the same conclusion must be drawn with respect to the other measurement of normative climate presented in Table 4: familialism does not affect male well-being gaps. For women, however, living in a familialistic society deepens the gap between those who are divorced and those who are married: a one standard deviation higher score on familialism goes together with an increase of 0.23 in the well-being gap (from −0.78 to −1.01). Theoretically, two interpretations of familialism seemed plausible. On the one hand, familialism expresses a norm that the family should be a strong entity; on the other hand, it implies that receiving support from family members is more likely. On the basis of the results in Model 4, the first interpretation seems to be the correct one. The level of well-being of divorced women compared to married women is lower as societies more strongly endorse familialism, presumably because divorced women feel the burden of having failed the norm of sustaining a family. A look at the contrast between single and divorced women (which is borderline insignificant with *p* = 0.07) confirms this interpretation. The fact that divorced women are, compared to single women, negatively affected by living in a familialistic society suggests that familialism does not imply more family support, as this would presumably affect women without a partner to the same extent. It suggests that divorced women suffer more strongly from the prescribed norm of having a strong family. After all, divorced women are likely to be considered (partly) responsible for breaking up a family, whereas single women have not actively acted against the norm of building a strong family, but have not met this norm yet.

Because the two value measures used in this study have a moderately high, positive correlation, it is sensible to test whether this finding is robust by including all interactions at the same time. This has been done in Model 5. The number of degrees of freedom becomes rather limited as 18 macro-level effects have to be estimated now, but nevertheless the mediating effect of familialism on the female married-divorced gap is not affected by the correlation between the two value measures, nor is any other effect.

## Conclusion

This study investigated the relationship between partnership status and subjective well-being with a special interest in the dependence of differences in subjective well-being on societies’ normative climates. I analyzed 45 countries studied in the European Values Study 2008, which contains detailed information on partnership status.

The first set of conclusions refers to the descriptive aim of this study. The results have shown that subjective well-being varies by partnership status, with married individuals reporting the highest level of well-being, followed (in order) by cohabiting, dating, single, and finally divorced or widowed individuals. Several remarks concerning this relationship can be made. Firstly, the order is generally in line with expectations based on associated differences in resources. Secondly, differences can partly be explained by household income and health, but the gaps remain substantial and in the same rank-order. Thirdly, some interesting differences between men and women emerged. Divorced women rank lower on well-being than widowed women—in line with the expected rank-ordering of well-being—whereas widowed men rank lower than divorced men. This cannot be explained by the fact that the drop in economic resources after divorce is larger for women than for men, as the difference remains after controlling for household income. Another interesting difference between men and women concerns the meaning of having a dating relationship. For men, being in a dating relationship brings significantly more well-being than being single. For women, it is not enough to have met someone; for them it is living together with a partner that makes the significant contribution to their well-being. Fourthly, although the difference in well-being between married and cohabiting persons is significant in the total sample, which contains many cases, it reaches the conventional level of significance in only 6 out of 45 countries. This does not unequivocally support the widely held belief of the existence of a marriage premium over-and-above cohabitation (Soons and Kalmijn 2009; Stack and Eshleman 1998). Other large-scale recent datasets can be used to test whether this finding can be replicated. Finally, the descriptive analyses demonstrate that, in studies on subjective well-being, current partnership status is a more appropriate operationalization of ‘marital status’ than legal marital status. Legal marital status masks heterogeneity among widowed, divorced or never married persons; these individuals are significantly happier if they have found a new partner with whom to date or cohabit.

The second set of conclusions concerns the impact of the normative climate on well-being gaps. The analyses revealed that gaps in well-being between partnership statuses vary over countries, with the exception of the marriage-cohabitation gap. However, country differences in well-being gaps are not the result of different normative climates in these countries. There is one exception to this general conclusion. Divorced women are more disadvantaged in terms of their subjective well-being than married women in familialistic societies that underline the value of a strong, close-knit family. This can be interpreted as divorced women in such societies perceiving their partnership status more as a failure to meet this family norm than divorced women in societies that place less emphasis on the family. Interestingly, the normative climate does not affect divorced men. Perhaps women are more affected by the norms and opinions of others than men, when assessing their degree of life satisfaction.

The general conclusion that can be formulated is that societies’ normative climates have little impact on how people in various kinds of relationships evaluate their lives. This conclusion may be interpreted as evidence in favor of the idea that in present-day society autonomy in private decisions, such as relationship-related decisions, is so high that people’s well-being is hardly affected by society’s norm regarding their partnership status. The next question then is whether the well-being of people in different partnership statuses is affected by practical issues in terms of the availability of resources outside the relationship, such as provisions from the welfare state or direct help by family and friends. Mulder et al. (2006) have argued and shown that resources are a less important determinant for first-union formation in conservative welfare states (the Netherlands and Germany) than in liberal welfare states (U.S.). This importance of the type of state may also apply to the impact of resources, which are related to partnership statuses, on subjective well-being. I suggest that this issue is examined further in future research.

A possible limitation of this study is that it cannot provide a thorough empirical assessment of the issue of selection. Previous literature has argued that the relationship between partnership and subjective well-being can be endogenous: high levels of well-being lead to a certain partnership status because happy people are more attractive (marriage) partners than unhappy people. Mastekaasa’s (1992) event-history analyses indeed showed that persons with higher levels of well-being are more likely to get married. However, the longitudinal study of Kamp Dush and Amato (2005) showed that well-being does not affect the likelihood of entering more committed relationships, for example when moving from dating to cohabitation, or from cohabitation to marriage. In addition, it is argued that unobserved heterogeneity can result in the observation of a spurious relationship between partnership and well-being: unmeasured characteristics affect both partnership status and well-being (e.g., Coombs 1991; Gove et al. 1990). Although one must be careful in interpreting the association between partnership status and subjective well-being reported in this study as entirely causal, Coombs (1991) concludes, on the basis of his extensive literature review, that selection is definitely not the driving force behind the observed relationship between marriage and well-being. One can argue that the ordering of partnership statuses according to the level of subjective well-being, found in this study, follows the prediction derived from the resources explanation, therefore making it plausible that at least some of the association is indeed causal. In addition, the selection issue is less relevant for the assessment of the moderating effect of the normative climate.

## Appendix

See Table 7.

**Table 7:** Distribution of partnership statuses by country

	Married	Cohabiting	Dating	Single	Widowed	Divorced
Albania	0.71	0.03	0.02	0.19	0.04	0.01
Armenia	0.63	0.01	0.02	0.20	0.09	0.03
Austria	0.48	0.11	0.08	0.19	0.06	0.07
Belarus	0.48	0.04	0.12	0.17	0.11	0.09
Belgium	0.61	0.08	0.09	0.12	0.04	0.05
Bosnia Herzegovina	0.58	0.02	0.09	0.19	0.04	0.03
Bulgaria	0.59	0.04	0.00	0.14	0.13	0.07
Croatia	0.54	0.04	0.08	0.18	0.06	0.03
Cyprus	0.67	0.02	0.03	0.14	0.09	0.04
Czech Republic	0.48	0.09	0.06	0.15	0.11	0.10
Denmark	0.60	0.14	0.05	0.11	0.03	0.06
Estonia	0.39	0.12	0.07	0.17	0.14	0.11
Finland	0.56	0.14	0.05	0.14	0.02	0.08
France	0.49	0.14	0.12	0.12	0.06	0.08
Georgia	0.66	0.00	0.02	0.17	0.12	0.04
Germany	0.53	0.09	0.06	0.14	0.07	0.10
Great Britain	0.49	0.09	0.05	0.16	0.08	0.12
Greece	0.62	0.02	0.07	0.15	0.09	0.04
Hungary	0.54	0.09	0.09	0.15	0.06	0.06
Iceland	0.65	0.06	0.05	0.13	0.01	0.09
Ireland	0.50	0.05	0.07	0.22	0.03	0.06
Italy	0.57	0.05	0.11	0.16	0.03	0.02
Latvia	0.54	0.06	0.06	0.12	0.10	0.09
Lithuania	0.52	0.06	0.05	0.16	0.12	0.08
Luxembourg	0.46	0.12	0.16	0.19	0.02	0.04
Macedonia	0.59	0.01	0.08	0.21	0.04	0.02
Malta	0.61	0.01	0.06	0.19	0.09	0.03
Moldova	0.64	0.04	0.04	0.12	0.12	0.05
Montenegro	0.53	0.03	0.09	0.23	0.07	0.05
Netherlands	0.66	0.05	0.05	0.11	0.07	0.06
Northern Cyprus	0.50	0.02	0.10	0.26	0.05	0.04
Northern Ireland	0.50	0.08	0.07	0.19	0.06	0.05
Norway	0.56	0.17	0.07	0.13	0.03	0.04
Poland	0.57	0.05	0.03	0.20	0.09	0.03
Portugal	0.62	0.03	0.03	0.14	0.10	0.06
Romania	0.64	0.06	0.04	0.10	0.11	0.04
Russian Federation	0.50	0.05	0.08	0.12	0.14	0.08
Serbia	0.57	0.04	0.08	0.15	0.09	0.06
Slovak Republic	0.62	0.04	0.02	0.10	0.13	0.05
Slovenia	0.62	0.04	0.07	0.15	0.06	0.02
Spain	0.52	0.05	0.08	0.21	0.07	0.07
Sweden	0.57	0.10	0.06	0.11	0.01	0.06
Switzerland	0.49	0.08	0.13	0.13	0.05	0.09
Turkey	0.73	0.01	0.02	0.16	0.05	0.02
Ukraine	0.59	0.05	0.04	0.10	0.13	0.09
Average	0.57	0.06	0.06	0.16	0.07	0.06
*N*	35,133	3,681	3,922	9,573	4,648	3,561

*Source*: European Values Study, 2008

## Footnotes

1 Kosovo appeared to be the only country in which widowed, single, and dating persons report significantly higher levels of well-being than married persons.

2 Missing values on household income were predicted on the basis of education, age, age squared, unemployment, and gender for each country separately. Missing values on education were predicted on the basis of age, gender, and employment status (distinguishing full-time, part-time, self-employed, unemployed, non-employed) for each country separately. The correlation between imputed income and observed income for respondents with non-missing values is 0.64; for education this correlation is 0.53. General drawbacks of mean imputations are that standard errors of estimates are too small and that associations can be distorted. Analysis on the sample with imputed values revealed the same results as analysis on a sample with simple country mean imputations; complete case analysis produces slightly different coefficients, but no different conclusions.

3 Additional analyses showed that the conclusions would not be different if the models would not control for the individual level interaction terms between values and partnership statuses.

4 Effect sizes are calculated as *b*(*x*)/sd(*Y*). For example, the effect size of the well-being difference between married and widowed men is 1.06/2.27 = 0.47. Standard deviations of the dependent variable are reported in Table 1.

5 Exact variance levels are 0.0000322 for men and 0.0000112 for women.

## References

[CR1] Bachrach C, Hindin MJ, Thomson E, Waite L (2000). The changing shape of ties that bind: An overview and synthesis. The ties that bind. Perspectives on marriage and cohabitation.

[CR2] Campbell A, Converse PE, Rodgers WL (1976). The quality of American life. Perceptions, evaluations, and satisfactions.

[CR3] Cherlin AJ (2004). The deinstitutionalization of American marriage. Journal of Marriage and the Family.

[CR4] Coombs RH (1991). Marital status and personal well-being: A literature review. Family Relations.

[CR5] Diener E, Gohm CL, Suh E, Oishi S (2000). Similarity of relations between marital status and subjective well-being across cultures. Journal of Cross-Cultural Psychology.

[CR6] Diener E, Suh EM, Lucas RE, Smith HL (1999). Subjective well-being: Three decades of progress. Psychological Bulletin.

[CR7] EVS. (2010). *European Values Study 2008, 4th wave, Integrated Dataset*. GESIS Data Archive, Cologne, Germany, ZA4800 Data File Version 2.0.0 (2010-11-30) doi:10.4232/1.10188. (10.4232/1.10188).

[CR8] Gove WR, Style CB, Hughes M (1990). The effect of marriage on the well-being of adults. Journal of Family Issues.

[CR9] Gundelach P, Kreiner S (2004). Happiness and life satisfaction in advanced European countries. Cross-Cultural Research.

[CR10] Kalmijn M (2007). Explaining cross-national differences in marriage, cohabitation, and divorce in Europe, 1990–2000. Population Studies.

[CR11] Kalmijn M (2010). Country differences in the effects of divorce on well-being: The role of norms, support, and selectivity. European Sociological Review.

[CR12] Kamp Dush CM, Amato PR (2005). Consequences of relationship status and quality for subjective well-being. Journal of Social and Personal Relationships.

[CR13] Kessler RC, Essex M (1982). Marital status and depression: The importance of coping resources. Social Forces.

[CR14] Kitson GC, Babri KB, Roach MJ, Placidi KS (1989). Adjustment to widowhood and divorce. Journal of Family Issues.

[CR15] Lane RE (2000). The loss of happiness in market democracies.

[CR16] Mastekaasa A (1992). Marriage and psychological well-being: Some evidence on selection into marriage. Journal of Marriage and the Family.

[CR17] Mastekaasa A (1994). The subjective well-being of the previously married: The importance of unmarried cohabitation and time since widowhood or divorce. Social Forces.

[CR18] McLanahan S, Sandefur G (1994). Growing up with a single parent. What hurts, what helps?.

[CR19] Mulder CH, Clark WAV, Wagner M (2006). Resources, living arrangements and first union formation in the United States, the Netherlands, and West Germany. European Journal of Population.

[CR20] Mynarska M (2010). Deadline for parenthood: Fertility postponement and age norms in Poland. European Journal of Population.

[CR21] Ongaro F, Mazzuco S, Meggliolaro S (2009). Economic consequences of union dissolution in Italy: Findings from the European Community Household Panel. European Journal of Population.

[CR22] Ross CE, Mirowski J, Goldsteen K (1990). The impact of the family on health: The decade in review. Journal of Marriage and the Family.

[CR23] Soons JP, Kalmijn M (2009). Is marriage more than cohabitation? Well-being differences in 30 European countries. Journal of Marriage and the Family.

[CR24] Soons JP, Liefbroer AC, Kalmijn M (2009). The long-term consequences of relationship formation for subjective well-being. Journal of Marriage and the Family.

[CR25] Stack S, Eshleman JR (1998). Marital status and happiness: A 17-nation study. Journal of Marriage and the Family.

[CR26] Wilson W (1967). Correlates of avowed happiness. Psychological Bulletin.

